# Supplementary material for: Are carbon emissions trading and green financial instruments synergistic? -Comprehensive quantitative research based on content analysis
Source: PLoS One. 2024 Mar 7;19(3):e0298601. doi: 10.1371/journal.pone.0298601 (PMC10919601; doi:10.1371/journal.pone.0298601)
Supplement: S1 Data — (ZIP) [file pone.0298601.s001.zip › Data/地级市Z_F工作报告.zip!/╝¬┴╓/░╫╔╜╩╨/└┤╫╘╣1⁄2╓┌║┼í╛┬φ┐╦╩2╛▌═°í┐_2017.docx]

各位代表：

　　现在，我代表市政府，向大会作政府工作报告，请予审议，并请市政协各位委员提出意见。

　　一、过去四年工作回顾

　　过去的四年，是白山转型发展极不平凡的四年。面对经济下行压力不断加大、转型升级刻不容缓、资源环境约束日益趋紧等多重挑战，在省委、省政府和市委的坚强领导下，我们团结带领全市广大干部群众，认真贯彻习近平总书记系列重要讲话精神，全面落实省委“三个五”及东中西区域战略部署，主动适应经济发展新常态，在抢抓机遇中干事创业、在迎接挑战中砥砺奋进，全力以赴稳增长、多措并举促转型，经济实力稳步提升、改革开放不断深化、社会事业繁荣进步、基础设施明显改善、民生福祉持续增进、生态建设成效显著。主要标志是“跃上两个新台阶、完成两个翻番、实现两个超越”：即2016年GDP和全口径财政收入分别跃上700亿元和70亿元台阶。民营经济主营业务收入1910亿元，旅游社会总收入125亿元，均比2012年翻一番。“新五样”占GDP和地方级财政收入比重分别达到61.5%和45.4%，超越“老三样”；城乡居民人均可支配收入年均增长8.8%和9.1%，超越GDP增速。这些成就的取得，标志着我市已经站在绿色转型新起点，正在集聚振兴崛起新能量。

　　刚刚过去的2016年，全市上下以市委“3331”战略为指引，以绿色转型为主线，以十大工程（战役）为统领，克难攻坚、精准发力，实现“十三五”良好开局。

　　— —经济发展势头强劲。预计实现GDP715亿元、地方级财政收入47亿元、服务业增加值258亿元，分别增长7%、5%和10%，增速始终居全省前列。扎实推进工业稳增长调结构增效益，实现规上工业增加值430亿元、增长7%，利润24.8亿元、增长11.7%。打响服务业发展攻坚战，推进46个服务业重点项目，服务业占GDP比重提高2个百分点。扩大有效投资，长白山鲁能胜地、市区地下综合管廊、西北岔水源地等一批重点产业、重大基础设施、重要民生项目顺利实施，全市开复工3000万元以上项目464个，完成投资438亿元。县域经济综合实力不断增强，GDP、规上工业增加值、地方级财政收入占全市总量比重分别达到73.7%、76%和72.9%，抚松、临江荣获全省县域综合发展（2类县份）二、三等优胜奖。

　　— —改革开放步伐加快。推进供给侧结构性改革，关闭退出煤矿26处，煤炭去产能484万吨，商品房去库存14万平方米，为企业减费降税4亿多元。加快“放管服”改革，建成责任清单，启动政务服务“一张网”建设，“五证合一”新登记企业3000户。加快投融资体制改革，组建江源、浑江农商行和长白榆银村镇银行，百亿级医药产业基金、新能源汽车产业基金落户白山，安广物流等3户企业上市。大力实施创新驱动战略，开展“百所高校进白山”、“院士专家白山行”等活动，新增健康服务业等3个院士工作站和林源春等6户国家高新技术企业。加快向东向南开放，长白国家重点开发开放试验区纳入新一轮振兴东北规划。出台招商引资22条意见，开展精准务实招商，借助台企吉林行、首届全球吉商大会、世界500强进吉林等平台全方位对接洽谈项目，成功引进安永、万景、银泰等一批战略投资者，引进域外资金增长10%。

　　— —城乡面貌显著改观。启动“六城联创”，全面综合整治城市环境。开工建设滨江等5条街路，拓宽改造浑江和长白山大街，金河大桥竣工通车；建成城区管廊管沟15.9公里、输水管线18公里，“气化白山”投入运营。城市景观进一步提升。栽植花卉150万株、乔木4300余株，新增绿地3.4万平方米；完成4条街路、23栋楼体亮化；改造3个老旧小区69栋居民楼。城镇化水平不断提高。统筹推进抚松新城建设、长白和靖宇旧城改造、临江引泉入城等工程；推进美丽乡村建设，高标准建设1个整体推进县、6个整体推进镇、36个示范村、8600个美丽庭院和2万户干净人家，松江河等5个镇入选全国重点镇。交通建设取得新突破。鹤大高速竣工通车，辉白高速全线开工，白临高速完成PPP模式实施方案，松江河至长白高速完成工可研，沈阳至佳木斯高铁前期工作加快推进，新机场项目列入“十三五”民航机场发展规划。

　　— —生态环境全面改善。开展环境保护专项行动，全面整治70项突出问题，完成减排项目8个、环保目标项目28个，取缔燃煤小锅炉30台，淘汰黄标车和老旧车3242辆，市区空气优良天数增加25天，松花江、鸭绿江、浑江干流出境水质达到3类以上标准，县级以上集中式饮用水源水质100%达标，超额完成主要污染物减排目标。推进生态红线区划定，完成珠宝河湿地公园等7个生态环境治理工程。清收林地12.8万亩，植树造林4.2万亩。建立矿山地质环境信息系统，实施11个地质灾害治理项目。健全绿色发展生态考核体系，推进生态环境资源公益诉讼，完成184户重点环境风险企业应急预案备案。

　　— —社会保持和谐稳定。高度重视安全生产工作，全力开展煤矿、道路交通、消防等重点领域大排查、大整改专项行动，深入落实国务院安委会第八巡查组反馈7方面21项整改措施，实现“五覆盖、五落实、五到位”，安全生产形势好转。群众饮食用药安全得到进一步保障。连续37年无重大森林火灾。健全矛盾纠纷多元化解工作格局，信访积案“百日攻坚”集中解决144件。扎实推进治安防控网建设，群众安全感满意度明显提升。强基富民固边成效显著，实现全国双拥模范城三连创。

　　— —民生福祉持续增进。全面打响三年脱贫攻坚战，落实各级包保责任，大力实施“五个一批”工程，推进194个产业扶贫和292个基础设施项目，脱贫6907人。改造各类棚户区38.8万平方米，货币化安置5381户、安置率100%。改造供热管网32公里、热力站8座，下调市区供热费。城镇新增就业4.4万人，城镇登记失业率4%，农村劳动力转移就业12.7万人。长白山职业技术学院新校区开工，职业技能培训公共实训基地顺利推进。文化综合体项目启动，“四馆”免费开放。基层医疗卫生机构全部实现基本药物零差率销售和网上集中采购，县级公立医院改革和农村卫生室标准化建设全面推进。出生人口性别比控制在110以内。短道速滑馆主体完工，新殡仪馆、养老服务示范中心、残疾儿童康复训练中心投入使用。民生实事除市区视频监控系统因主街路拓宽改造未如期实施外，其余25件全部完成。

　　— —民主法治和政风建设进一步加强。扎实推进依法行政，出台重大行政决策程序规定，开展全省行政大复议试点，推广“双随机一公开”监管。扎实开展“两学一做”学习教育，全面完成省委第二轮巡视“回头看”反馈12项整改任务。加强廉政建设，实施“权力入笼”工程，强化审计、软环境等监督，推进政务督查、跟踪问效，推动各项决策部署落地生根。自觉接受人大、政协和社会各界监督，办理人大代表建议和政协委员提案124件，面复率、满意率均达100%。

　　各位代表，这些成绩的取得，是省委、省政府和市委坚强领导的结果；是市人大、市政协和社会各界监督支持的结果；是全市人民开拓创新、共同奋斗的结果。在此，我代表市政府向全市各族人民，向驻白山人民解放军指战员、武警部队官兵、公安司法干警、中省直单位，向各民主党派、工商联、人民团体、无党派人士、离退休老同志，向关心支持白山发展的各界朋友，致以崇高的敬意和衷心的感谢！

　　在总结成绩的同时，我们也清醒认识到存在的差距和问题：一是经济总量不大，综合实力不强，财力不能满足经济社会发展需求，民生保障水平不高；二是转方式、调结构、促转型任务依然繁重，新旧动能转换仍需持续发力，“老三样”向“新五样”转型仍需提档加速；三是向东向南开放需要新突破，落实“三去一降一补”任务仍需加大力度；四是城市品位不高、承载力不强的问题还未得到有效解决，生态建设和环境保护压力较大，与宜居宜业宜游目标有较大差距，土地征收、开发区建设以及融资等发展瓶颈制约严重；五是部分政府工作人员发展意识、大局意识、责任意识不强，不作为、乱作为，不敢担当、不善担当现象时有发生等。对此我们将采取有力措施，认真加以解决。

　　二、未来五年目标和任务

　　未来五年，是白山绿色转型全面振兴的关键期，全面建设小康社会的决胜期，更是发展优势充分释放、新旧动能加速转换、改革开放活力迸发、城乡建设协调推进、人民群众幸福感和获得感显著提升的战略机遇期。市政府工作的总体要求是：高举中国特色社会主义伟大旗帜，以邓小平理论、“三个代表”重要思想和科学发展观为指导，深入贯彻党的十八大，十八届三中、四中、五中、六中全会和习近平总书记系列重要讲话精神，牢牢把握“五位一体”总体布局、“四个全面”战略布局、五大发展理念、稳中求进工作总基调，按照省委“三个五”战略部署，认真贯彻落实市第七次党代会精神，坚持生态立市、产业强市、特色兴市不动摇，深入实施“3331”战略，全力打造吉林东部绿色转型发展先行区、国家生态文明建设示范区，加快白山绿色转型全面振兴，与全省同步全面建成小康社会。

　　未来五年发展的奋斗目标是：

　　— —经济质量更加优化，转型振兴卓有成效。全力打造全省东部绿色转型发展先行区，到2021年实现“两个高于、两个突破、三个跃升”目标，即GDP和城乡居民人均可支配收入增速高于全省平均水平，GDP和财政收入分别突破千亿和百亿级大关，“新五样”、服务业和民营经济占GDP比重分别跃升至70%、46%和75%以上。

　　— —城乡统筹更加协调，一体化格局深入推进。全力推进城乡统筹协调发展，中心城市品位和承载力显著提升、县城建设迈上新台阶、重点城镇彰显特色、农村面貌明显改观，基础设施全面改善，基本形成资源共享、优势互补、布局合理、特色鲜明的城乡发展新格局，建成宜居宜业宜游的长白山区域生态城市。

　　— —发展活力更加充沛，创新体系日趋成型。全力实施创新驱动发展战略，以科技创新为支撑的经济体系和发展模式初步形成。供给侧结构性改革全面落实，供给体系质量和效益显著提高，“放管服”等重点领域改革取得决定性成果，市场主体活力充分激发。全面融入国家“一带一路”和我省向东向南开放两翼并进战略，着力打造鸭绿江开发开放经济带，形成“东西互济、南北互通、多向开放”新格局。

　　— —人与自然更加和谐，生态文明成为示范。全力打造国家生态文明建设示范区，到2021年实现“一个领先、四个更加”目标，即生态环境质量全省领先，生态空间格局更加优化、生态安全屏障更加稳固、生态资源优势更加突出、生态承载功能更加完善。主要污染物排放量持续减少，产业低碳化、交通清洁化、建筑绿色化、可再生能源利用规模化水平大幅提升，绿色生态理念和低碳环保生产生活方式更加普及。

　　— —人民生活更加幸福，公共服务体系日益完备。全力建设和谐、平安和法治白山，城乡居民收入大幅增加，贫困人口全部脱贫，就业更加充分，社会保障全覆盖，社会事业全面进步，公共服务体系更加健全，社会治安和安全生产形势持续向好，边境安全稳定。

　　围绕上述目标，新一届政府将重点抓好五方面工作：

　　（一）充分释放发展优势，加快产业转型升级。着力推动“老三样”、“新五样”向集聚化布局、规模化发展、中高端迈进，将生态、资源等优势转化为竞争、发展优势。大项目支撑。全力以赴实施一批成长性、创新性、引领性强的大项目好项目，着力在优化存量、突破增量、提升质量上下功夫，为产业转型夯实基础、积蓄动能。大企业带动。鼓励企业开展资本运作和强强联合，不断发展壮大百亿龙头、十亿骨干、亿元规模企业梯队，形成一批国内一流、东北领军的行业旗舰，力争五年30户以上企业上市。大品牌驱动。大力实施品牌战略，发扬“工匠精神”，培育“百年老店”，争创一批中国名牌产品和驰名商标，打响长白山矿泉水、人参和旅游等国内同行业“第一品牌”。大产业集聚。围绕龙头企业、主导产品，不断细化产业分工协作，搞好上下游产业衔接，形成关联度紧密的产业链，构建一批发展层次高、协同效应好、配套服务优、特色优势强的百亿级产业集聚区。全力建设国家级新材料基地、国际矿泉城、世界人参之都、长白山北药生产研发基地、国家全域旅游示范区和长白山区域现代服务业中心。

　　（二）坚持创新驱动，加快新旧动能转换。牢固树立“抓创新就是抓发展、谋创新就是谋未来”理念，释放发展新动能。打造一批 “双创”基地。放宽政策、放活市场、放开空间，激发全社会创新潜能和创造活力，让大众创业、万众创新蔚然成风。建设一批创新平台。完善厅市会商和政产学研用合作机制，鼓励企业与高校院所共建高层次创新载体，加快组建多领域院士工作站、博士后工作站、产业技术创新联盟、企业研发中心和工程技术中心。到2021年，全社会研发经费支出占GDP比重超过1.6%，每万人拥有发明专利1.5件。推进一批创新项目。在硅藻土、生物医药等领域掌握一批行业领先和核心关键技术，打通科技成果转化通道，有效对接创新链和产业链。到2021年，规上工业增加值中高新技术产业比重达到16%以上。培育一批创新人才。推进重大人才工程、人才计划和人才培养项目，建设长白山职业技术学院“万人大学”，引进高水平创新团队，培养实训型、实用型、实践型人才，为创新发展提供强大智力支撑。扶持一批创新企业。引导科技型企业不断提升创新能力，实施“小升高、小壮大、小做强”工程，形成一批科技型小巨人企业。到2021年，国家高新技术企业达到18户以上，创新型科技企业36户以上。

　　（三）深化改革开放，加快向东向南开放步伐。将深化改革、扩大开放作为绿色转型的关键之举和必由之路。打好供给侧结构性改革攻坚战。全面落实“三去一降一补”，完成煤炭去产能任务；有效化解房地产库存，促进房地产业持续健康发展；增强金融服务实体经济能力，防范区域性金融风险；综合施策降低企业成本，优化生产经营环境；聚焦脱贫攻坚、基础设施、公共服务等领域，加快补齐发展短板。深化重点领域改革。推进以“放管服”为重点的行政管理体制改革，加快转变政府职能。积极争取扩权强县、扩权强镇试点，承接和下放好经济管理权限。稳步推进国有林场改革，争创森工城市经济转型和林区生态移民试点，发展碳汇经济。推进就业创业、社会保障、医疗卫生等公共服务领域改革，加快科技、财税、土地等配套改革，进一步深化农村综合改革。全方位扩大开放。深入落实两翼并进和“南联东拓北接”开放总要求，以鸭绿江开发开放经济带为载体，加快白通丹一体化，连接环渤海经济圈和长吉图开发开放先导区，扩大对外贸易，加强与东北东部城市群互通联动，打造沿边开发开放新增长极。高水平推进招商引资。坚持走出去请进来并重、引资引智引技并举，主动对接江浙沪等重点区域，推进世界和国内500强、央企、民企进白山，提高利用域外资金、人才、技术质量和水平。大力发展总部经济和楼宇经济，引进一批大企业在白山设立总部或分支机构。加快开发区转型升级、创新发展，建设一批基础设施完备、集聚效应明显、经济成长性好的示范园区。

　　（四）大力推进新型城镇化，加快城乡统筹发展。有序推进以人为核心的新型城镇化，在强化发展协调性中壮大整体实力。做优中心城市。加强城市规划、建设、管理，编制生态城市总体规划和专项规划，推进“多规合一”，加快“北跨南控、东拓西进”，实施“净畅绿亮美”五化工程，形成“四街四路四出口”城市特色风貌轴，“一江十河”城市滨水景观廊道，“一山一湖”城市后花园。集聚人口、土地等要素资源，提升中心城市功能。做强县城。全面推动县城扩容提质，增强辐射带动能力，打造功能完善、产业集聚、生态宜居县城。做精重点城镇。突出一镇一特色、一镇一风貌，创建一批旅游观光、现代农业、新型工业、商贸物流、生态园林型城镇，争创国家级新型城镇化试点和特色小镇。做美宜居乡村。实施“一十百千万”工程，打造200个高标准美丽乡村示范村，共建共享“一村一景、一村一业、一村一韵”特色乡村。做大交通体系。辉白高速通车，加快建设白临、松长高速和鸭绿江边防公路，推动国省干线和农村公路建设，形成“四高速、两高铁、两机场”为骨架的“六横七纵两口岸”立体化大交通体系。

　　（五）坚持生态立市不动摇，加快生态文明建设。始终坚持在保护中发展、在发展中保护，筑牢绿色屏障，发展绿色产业，践行绿色生活，共建绿色家园。争创全国最优生态环境。全面落实主体功能区规划，严格实行空间、总量、项目“三位一体”环境准入制度，严守生态红线。做好“治山理水、显山露水”文章，实施“三江源”山水林田湖生态保护和修复，建设“三江一山”生态廊带，打好大气、水体、土壤污染防治攻坚战，加强自然保护区和野生动植物栖息地管护，让青山常在、绿水长流、空气常新。全市森林覆盖率达到84.5%以上，城市绿化率达到40%以上，市区空气优良天数达到300天以上。大力发展生态经济。严控“三高”行业发展和低水平重复建设，引导技术和资金更多投向绿色产业。发展低碳循环经济，推行企业循环式生产、产业循环式组合、园区循环式改造，推进工业“三废”综合治理和回收利用，提高城乡垃圾无害化、减量化、资源化、一体化处理水平。全面建设“两型”社会。坚持节约优先，推动资源利用方式根本转变，落实全民节能行动计划，实行最严格的水资源管理、耕地保护和林木禁伐制度。健全生态资源保护管理体系，探索建立自然资源资产产权、用途管制和生态补偿制度。完善生态文明考评机制，建立领导干部生态文明建设责任制。树立尊重自然、顺应自然、保护自然的生态理念，倡导勤俭节约、低碳绿色、文明健康的消费模式，使“绿色化”成为人民群众的自觉行动。

　　各位代表！通过五年的不懈努力，一个经济实力更强、开放层次更高、生态环境更优、区域发展更协调、人民生活更幸福的新白山，将在吉林东部振兴崛起！

　　三、2017年工作安排

　　2017年是新一届政府的开局之年，是全面贯彻落实市第七次党代会精神的第一年，也是我市绿色转型全面振兴承上启下的重要一年。今年全市经济社会发展的预期目标是：GDP增长7.5%，规上工业增加值增长6%，服务业增加值增长12%，地方级财政收入增长5%，固定资产投资增长8％，社会消费品零售总额增长10%，城乡居民人均可支配收入与经济增长同步，城镇登记失业率控制在4.5%以内，单位GDP能耗降低3.2%，碳排放强度下降3.96%。

　　实现上述目标，要突出抓好九方面重点工作：

　　（一）坚定不移稳增长。坚持统筹推进，对症施策，增强调控精准性，确保经济稳定增长。促进工业经济提质增效。深入开展工业经济“保增长、促发展、增效益”攻坚活动，实施重点企业“一对一”精准帮扶，健全运行监测、科技信息、电子商务、融资服务“四大平台”。积极争取睿弛通航旋翼机、鑫霖超纯二氧化硅微粉等135个项目列入省“五个一批”计划。集中力量推进服务业攻坚。加快建设综合物流枢纽中心和靖宇、松江河物流园区，推进宽带中国示范市和智慧城市建设，做强电商进农村2个国家级、1个省级示范县，确保江源、长白列入省级示范，在全省率先实现示范县全覆盖，推动生产性服务业向专业化、现代化延伸。推进昌泰怡康颐养中心等项目，扶持欧亚、方大等商贸流通企业发展壮大，进一步繁荣消费市场、促进消费升级，推动生活性服务业向精细化、高品质转变。大力发展县域经济。打造人参、北药、食用菌等一批特产基地，做强大山合、同仁堂等一批龙头企业，建设一村一品示范村镇。推进广泽度假区、葵花药业临江健康产业园、天安镁业二期、长白加一人参、修元200万吨矿泉水等重大产业项目。强化县域经济支撑，努力实现争先晋位。着力振兴实体经济。开展质量提升行动，完善质量标准体系，推进消费品工业“三品”专项行动。实施中小微科技企业培育工程，加快取得一批突破性科研成果，尽快形成现实生产力。加大政银企对接力度，加强企业人才队伍建设，帮助企业开拓销售市场。落实减费降税政策，实施降本增效行动，多措并举降低企业成本。

　　（二）坚定不移调结构。实施产业转型攻坚计划，加快构建具有白山特色的绿色产业体系。做大做强“新五样”。深入实施找矿突破行动，力争147个探矿权早出成果，小栗子金及多金属矿详查等5个项目提交地质报告。推进远通硅藻土深加工、玄武岩拉丝等项目，深度挖掘矿产资源潜能，提升附加值和竞争力。推动农夫山泉、泉阳泉扩能提效，开发中高端产品。抓好人参生命科技产业园、1500万支小容量注射剂等项目，大力支持施慧达药业投产新品种，努力培育我市第一个产值百亿元、税收10亿元的龙头企业。积极构建以“冰雪旅游、冰雪体育、冰雪文化”为核心的“3+X”冰雪全产业链，强力推进长白山国际度假区做精做优、做大做强，确保鲁能胜地运营达效。接待域外游客增长15%，旅游社会总收入增长20%。改造提升“老三样”。坚持“改造、提升、扶优、劣汰”，重点支持通矿集团、板石矿业、浑江电厂、六大国有森工局等企业由单一资源生产向多元经营转换，使煤林铁等产业“老树发新枝”。培育战略性新兴产业。聚焦“中国制造2025”战略，着力发展新能源、节能环保、生物经济、文化创意、互联网+、大数据等产业，推进年产5万辆新能源汽车等项目，打造经济增长新引擎。

　　（三）坚定不移抓好重大项目建设和招商引资。大力开展“冬季三项促投资活动”，实施3000万元以上项目342个。强化投资拉动。集中推进新开工项目前期工作，切实解决项目审批、资金落实、土地征收等问题，加快安装调试设备、办理投产手续、落实建设条件，推动早落地、早开工、早见效。加快推进50万吨瓦楞纸等152个新建项目和舜茂高纯二氧化硅等190个续建项目，竣工投产春柏药业等60个项目。强化招商引资。变冬闲为冬忙，打好“冬春会战”，健全“五个一”工作机制，围绕“新五样”、战略性新兴产业、存量企业、重大项目和重要战略投资者以及补短板，做好有中生新、无中生有文章，开展精准务实招商。建立市县两级招商引资项目库，完善考核办法，提高招商实效。积极组织参加东博会和吉商大会，筹办矿产新材料和人参医药产业高峰论坛，组织开展珠三角、长三角知名企业进白山等活动。引进域外资金增长9%。强化开发区建设。统一规范土地征收，盘活挖潜存量，实施净地招商。完善配套设施，尽快达到“七通一平”。将各级开发区和工业集中区作为经济发展的主阵地和排头兵，积极推动项目集聚、要素集中，促进经济体量、承载容量、发展质量跃上新台阶，搭建高标准项目承载平台。

　　（四）坚定不移深化改革创新。加快体制机制改革，进一步释放改革红利，激发创新活力。全面推进供给侧结构性改革。推进煤矿去产能，妥善安置分流人员，关闭退出15处煤矿，去产能135万吨；加大房地产去库存力度，棚改货币化安置率和去库存率分别达到90%和10%；积极稳妥推进实体经济去杠杆，实施百户企业上市培育工程，完善上市企业优惠政策，新培育林源春等2户企业上市，企业直接融资比增长10%；设立基础设施建设基金，构建政府统一融资平台。深入推进农业供给侧结构性改革。发展生态效益型特色农业，加快农业现代化。调整农业种植结构，狠抓农产品标准化生产、品牌创建、质量安全监管，实施放心农产品质量安全工程，建立可追溯体系，主要农产品综合检测合格率95%以上；推进“农业+旅游”新模式，争创国家级休闲农业示范区，广辟农民增收致富门路；依托渤商所、邮乐农品网等平台，推进互联网+特色农业，增加绿色优质农产品有效供给；深化农村金融改革，完善金融服务体系；推进“三权分置”，完成农村土地确权登记颁证。提升科技创新能力。持续开展“百所高校进白山”、“院士专家白山行”等活动，强化企业与高校院所合作交流。加大创新平台建设力度，成立中国人参研究院，建设省科技大市场白山分市场。继续实施“引进名校优生”计划，培育创新人才队伍。大力发展民营经济。按照“事前引导、事中帮扶、事后奖补”原则，落实健全各项支持政策。鼓励民企二次创业，加快制度、科技、管理和商业模式创新。培养引进现代企业家、职业经理人和专业人才。推进“互联网+民营经济”，引导更多民企利用信息技术加速成长。争创首批东北地区民营经济发展改革试点。民营经济主营业务收入增长7.3%。

　　（五）坚定不移扩大开放。搞好顶层设计，高标准规划建设西起浑江三道沟，经江源、临江（靖宇）、长白至抚松松江河，总长500公里的鸭绿江开发开放经济带，举全市之力构建绿色转型振兴平台、改革开放创新平台、兴边富民固防平台，以开放促发展、促振兴。要加大基础设施建设力度，加快鸭绿江边防公路和“两高速、两高铁、一机场”建设，促进互联互通，打造我省向东向南开放的连接带。要启动鸭绿江中朝国际旅游风情带建设，整合全市旅游资源，串珠成链，打造世界闻名、国内一流、东北典范的旅游产业带。要抢抓机遇、争取支持，谋划包装一批大项目，打造国家和省各项政策的承接带。要推进长白国家重点开发开放试验区，长白、临江边境经济合作区，十个开发区以及各类特色园区建设，强化开放载体，形成开放合力，打造带动各县（市、区）加快发展的辐射带。

　　（六）坚定不移抓好城乡建设。坚持规划先行，完成城市总体规划和城市设计等十大类专项规划，持续加大投入，改善城乡面貌。提升城市品位和承载力。扎实推进36项重点任务109项具体任务。抓好净化：开展私搭乱建、城市牛皮癣、违章占道等专项整治，重点改善城乡结合部、背街小巷等环境薄弱地段“脏乱差”面貌。打造长白山大街等14条标准化示范街路，机械化清扫率达到85%。淘汰25台小锅炉。实施金坑河等4条黑臭水体整治。抓好畅化：打通6条断头路，改扩建14条街路、2座危桥，改造人行步道26.8万平方米。抓好绿化：大力增加城市绿量，移栽大树1万株以上。对广场、绿地、街角空地进行绿化升级改造和见缝插绿，提升浑江两岸绿化景观，改造绿地5.9万平方米，启动建设长白山特色植物园。抓好亮化：以一江两岸为中心，突出品位特色，完成90栋楼体、4个广场亮化，为39条无照明街路安装路灯。抓好美化：改造升级3个城市出入口，完成临街109栋楼房“穿衣戴帽”。改造13个老旧小区207栋居民楼。推进地下综合管廊等重点工程建设，统筹各类管线入廊。治理“一江十河”，打造亲水慢行系统。加快城市管理执法体制改革，下移管理重心，实施网格化精细管理，提高城市治理能力。加快城镇化步伐。完成抚松镇9条市政道路、靖宇镇污水管网和长白镇二次供水改造、临江新市街道地下管廊等一批市政基础设施工程，启动抚松镇江堤“慢行系统”、临江新市街道江滨公园建设和长白镇体育场、博物馆改造，建成靖宇镇河堤路景观带。推进美丽乡村建设。高标准建设22个省级重点村和36个美丽示范村，打造5千个美丽庭院、1万户干净人家。完成100个村环境综合整治。打造民俗文化、边境风情、生态宜居村庄，形成“一村一景致、一派好风光”新风貌。加快重大交通基础设施建设。加快建设辉白高速，推进白临、松长高速前期工作。白山至三道沟公路全线贯通，三道沟至错草公路开工，启动市区南外环一级公路前期工作，新建农村公路136公里。加快推进白山经通化、四平至长春高铁前期工作，开工建设沈阳至佳木斯高铁。完成新机场预可研编制。

　　（七）坚定不移抓好生态建设和环境保护。牢固树立“绿水青山就是金山银山”理念，启动创建国家生态文明建设示范区。加大生态保护力度。实施山体、水系、森林等生态修复工程，治理水土流失15万亩，人工造林1.2万亩，森林抚育1.9万亩。推进绿色矿山建设，实施8个矿山环境恢复治理项目。编制《三江源综合整治规划》，推进山水林田湖保护修复，积极谋划包装工矿废弃地复垦、矿山地质环境恢复治理等项目，加大地质灾害防治力度。实施最严格的自然生态保护制度，健全生态红线划定区域管控措施和国土空间用途管制制度。开展人居、田园等各类生态示范创建活动。全面落实“三区”整改任务，确保生态系统稳定性明显加强，生物多样性下降趋势得到有效遏制。加大环境保护力度。落实“党政同责、一岗双责”，开展环境问题综合整治，实施清洁空气、水体、土壤和生态建设行动，全面落实39项重点工作98项具体任务。启动“河长制”，推进浑江“母亲河”和大气污染等专项治理，市区空气优良天数达到290天以上，继续保持重点流域水质3类以上标准，集中式饮用水源地水质全面达标，农用地和建设用地土壤环境安全得到基本保障。

　　（八）坚定不移抓好平安白山建设。紧紧围绕安全发展、社会稳定和长治久安目标，努力建设更高水平的平安城市。突出抓好安全生产。坚持发展决不能以牺牲人的生命为代价，全面落实“五个责任、五大体系”，开展“五个一工程”。突出煤矿安全重中之重，加强正常生产煤矿监管监察、杜绝整改煤矿非法生产、确保退出煤矿关闭到位。强化非煤矿山、道路交通、消防等重点领域安全监管。严格执行一票否决和责任追究制，从严打击违法违规行为，有效遏制各类事故。实施食品药品放心工程，推进食品检验检测中心建设。实现连续38年无重大森林火灾。突出抓好社会治理。健全矛盾纠纷排查化解机制，开展“重点信访案件化解年”活动，做到“事要解决、案结事了”。建设立体化、信息化治安防控体系，开展打击盗抢骗、非法集资等专项行动。推进“七五”普法，加强法律援助、社区矫正、戒毒执法工作。依法加强宗教管理，切实维护民族团结。突出抓好边境安全。深化“兴边富民”行动，强化党政军警民合力治边。加强优抚安置，巩固双拥共建成果。

　　（九）坚定不移抓好民生和社会事业发展。进一步织密扎牢民生保障网，办好九大方面37件民生实事。扎实推进脱贫攻坚。以产业培植推动“造血扶贫”，实施产业脱贫项目200个以上。推进“五提高、一降低、一增加”健康扶贫工程，保障农村贫困人口享有基本医疗卫生服务。继续推进教育扶贫全覆盖行动。深化市直部门包村、干部包户全覆盖，引导社会扶贫重心下移，构建大扶贫格局。全年脱贫1万人以上，浑江、江源、临江完成脱贫任务。进一步完善社会保障体系。城镇新增就业4.2万人，城镇登记失业率控制在4.5%以内，农村劳动力转移就业12万人。扩大五大险种覆盖面，推行城乡居民医保“六统一”，社保“一卡通”覆盖率达到60%。提高社会救助水平，城乡低保标准分别达到上年度城乡居民人均可支配收入的20%和30%。加快建设康健医院、残疾人康复中心。改造城市和国有工矿棚户区33万平方米。全面发展各项社会事业。深化教育改革，完成全市县域义务教育均衡发展国家级评估验收，打造中等职业教育省级示范实训基地和示范校，加快建设长白山职业技术学院新校区。围绕健康白山建设，建立重大疾病“三位一体”防控机制，建设市中心医院全科医生临床培养基地。强化计生工作，促进人口均衡发展。提高“四馆”服务水平，建设城市文化综合体，创建国家级公共文化服务示范项目。大力发展竞技体育和全民健身，举办市第八届运动会。

　　四、加强政府自身建设

　　以加快职能转变为核心，不断加强作风和能力建设，增强执行力和公信力，努力打造人民满意政府。

　　一是强化法治思维，打造法治政府。始终把依法行政贯穿政府工作全过程，严格履行重大行政决策程序，建立终身责任追究和倒查机制。配合做好城市管理综合执法、物业管理、市容和环境卫生方面立法工作。推进全国重大执法决定法核试点建设，巩固行政大复议和争议调处双试点成果，健全行政裁决工作机制，促进规范、公正、文明执法。自觉接受人大法律监督、工作监督和政协民主监督，主动接受司法监督、社会监督和舆论监督，认真办好人大代表建议和政协委员提案。

　　二是强化便民利民，打造服务政府。坚持“放管服”多管齐下，协同推进，确保接住用好上级下放事项，推进权力真正下放到位。建成“四单一网”，实现一号申请、一口受理、一网办结，构建“互联网+政务服务”体系。健全政府购买公共服务机制，支持更多行业协会、社会组织参与公共产品和服务供给。建立重大项目审批“绿色通道”和“一条龙服务制”，实行“首办负责、并联审批、全程代办、限时办结”，确保工业项目在35个工作日内、房地产等基础设施类项目在55个工作日内完成所有审批事项。

　　三是强化信用建设，打造诚信政府。开展优化投资营商环境专项行动，加强各种所有制经济产权保护，完善政府守信践诺机制。深入推进政务决策、执行、管理、服务、结果公开，让行政权力在阳光下运行。完善社会信用体系，探索实行“互联网+监管”模式，建立健全市场主体诚信档案、行业黑名单制度和市场退出机制，加大对失信行为约束惩戒力度，推进信用白山建设。

　　四是强化责任担当，打造效能政府。进一步解放思想、开阔视野、奋发有为、善于担当，提升工作水平和标准。坚持一张蓝图绘到底，谋实策、出实招、办实事、求实效，以“钉钉子”精神狠抓各项工作落实。对决策部署定时间、定标准、定责任，做到件件有着落、事事有回音、项项有结果。加强政务督查和绩效考核，全面落实效能监察、行政问责等制度，坚决整治不作为、慢作为、乱作为等问题。健全正向激励、容错纠错机制，树立务实重行、干事创业导向。

　　五是强化从严治政，打造廉洁政府。不断增强“四个意识”，认真执行《准则》和《条例》，全面落实“两个责任”和“一岗双责”，从重点领域、重点环节入手，完善惩治和预防腐败体系，加大涉软和腐败案件查处力度。进一步加强监察工作，强化项目招标、政府采购、土地出让等专项审计，建立统一的公共资源交易平台，从源头上防止腐败。驰而不息纠正四风，严控政府系统三公经费支出。

　　各位代表！新形势、新机遇、新起点，赋予我们新使命、新责任、新担当。宏伟蓝图已经绘就，未来征程正在开启。我们有决心、有信心更加紧密地团结在以习近平同志为核心的党中央周围，在省委、省政府和市委的正确领导下，以更加高昂的斗志、更加务实的作风、更加扎实的举措，为加快白山绿色转型全面振兴而努力奋斗！
